# Reducing physical and emotional violence by teachers using the intervention Interaction Competencies with Children – for Teachers (ICC-T): study protocol of a multi-country cluster randomized controlled trial in Ghana, Tanzania, and Uganda

**DOI:** 10.1186/s12889-021-11950-y

**Published:** 2021-10-24

**Authors:** Florian Scharpf, Anette Kirika, Faustine Bwire Masath, Getrude Mkinga, Joseph Ssenyonga, Emmanuel Nyarko-Tetteh, Mabula Nkuba, Amoah Kwaku Karikari, Tobias Hecker

**Affiliations:** 1 Department of Psychology, Bielefeld University, Bielefeld, Germany; 2 Department of Educational Psychology and Curriculum Studies, Dar es salaam University College of Education, Dar es Salaam, Tanzania; 3 Department of Educational Foundations and Psychology, Mbarara University of Science and Technology, Mbarara, Uganda; 4 Presbyterian University College, Abetefi, Ghana

**Keywords:** School violence, Teacher violence, Intervention, Teachers, Students, Primary schools, Secondary schools

## Abstract

**Background:**

Violence has severe and long-lasting negative consequences for children’s and adolescents’ well-being and psychosocial functioning, thereby also hampering communities’ and societies’ economic growth. Positive attitudes towards violence and the lack of access to alternative non-violent strategies are likely to contribute to the high levels of teachers’ ongoing use of violence against children in sub-Saharan African countries. Notwithstanding, there are currently very few school-level interventions to reduce violence by teachers that a) have been scientifically evaluated and b) that focus both on changing attitudes towards violence and on equipping teachers with non-violent discipline strategies. Thus, the present study tests the effectiveness of the preventative intervention Interaction Competencies with Children – for Teachers (ICC-T) in primary and secondary schools in Tanzania, Uganda, and Ghana.

**Methods:**

The study is a multi-site cluster randomized controlled trial with schools (clusters) as level of randomization and three data assessment points: baseline assessment prior to the intervention, the first follow-up assessment 6 months after the intervention and the second follow-up assessment 18 months after the intervention. Multi-stage random sampling will be applied to select a total number of 72 schools (24 per country). Schools will be randomly allocated to the intervention and the control condition after baseline. At each school, 40 students (stratified by gender) in the third year of primary school or in the first year of secondary/junior high school and all teachers (expected average number: 20) will be recruited. Thus, the final sample will comprise 2880 students and at least 1440 teachers. Data will be collected using structured clinical interviews. Primary outcome measures are student- and teacher-reported physical and emotional violence by teachers in the past week. Secondary outcome measures include children’s emotional and behavioral problems, quality of life, cognitive functioning, academic performance, school attendance and social competence. Data will be analyzed using multilevel analyses.

**Discussion:**

This study aims to provide further evidence for the effectiveness of ICC-T to reduce teacher violence and to improve children’s functioning (i.e., mental health, well-being, academic performance) across educational settings, societies and cultures.

**Trial registration:**

The trial was registered at clinicaltrials.org under the ClinicalTrials.gov identifier NCT04948580 on July 2, 2021.

## Background

The ratification of the United Nations’ Convention on the Rights of the Child and the adoption of the Sustainable Development Goals document the international community’s efforts to protect all children from any form of violence in their environment. Notwithstanding, it has been estimated that more than 300 million children (17.5% of all children worldwide) experience severe forms of violence during their upbringing [1]. In school settings, where children and adolescents spend most of their time apart from their families, they may also face violence by school staff [2]. Teachers frequently use different acts of physical and emotional violence against children in order to regulate or correct misbehaviour [3]. These violent disciplinary measures may include beatings with the use of hands or objects, such as a cane or stick, shaking, pinching or kicking students, forcing them to adopt painful bodily postures for a long time as well as public humiliation [4, 5]. Such violence by teachers inflicts severe physical and emotional suffering and pain on children and may adversely affect their mental health, psychosocial functioning and academic achievement [6–8].

### Global perspective on violence against children by teachers

Violence against children and adolescents in schools is a global problem, which is illustrated by the fact that the use of physical violence by teachers is legally accepted as a disciplinary measure in 64 countries worldwide, mostly low- and middle-income countries in Africa and Asia [9]. Systematic reviews indicate high lifetime prevalence rates of more than 70% and up to 100% for physical violence by teachers in low- and middle-income countries, particularly in sub-Saharan Africa [5, 10]. Notably, prevalence rates were also high in countries where physical violence in schools is unlawful, suggesting that a legal ban may be a necessary, but not sufficient condition for ending the use of violence against students. The reliance on small, non-representative samples and cross-sectional assessments as well as the lack of rigorous methods are noted as major limitations of available prevalence studies [10]. Similar to physical violence, studies from various countries including Turkey, South Korea, Bangladesh, Uganda, Tanzania and Nigeria reported high rates of emotional violence by teachers ranging from 18% up to 100% [6, 8, 11–14].

### Factors contributing to teachers’ use of violence against children in sub-Saharan Africa

The consistently high prevalence of physical and emotional violence by teachers in primary and secondary schools in sub-Saharan African countries can be attributed to multiple structural, institutional, community, interpersonal and individual factors, which interact in a complex and dynamic manner [15, 16]. A legal framework may deter teachers from using violence against students due to fear of repercussions. However, social norms, beliefs and approval from authority figures condoning the use of violent discipline by parents, teachers and other adults in the community to educate children are particularly widespread in many societies in sub-Saharan Africa [17]. Accordingly, quantitative and qualitative data from various African countries suggests that teachers perceive violent discipline as an effective and acceptable way to exercise power over, enforce discipline and instill respect among students, but also to motivate them and foster learning opportunities [18–20]. Positive attitudes towards violence have been shown to mediate the association between African teachers’ stress [21] as well as their own experiences of violence [22] and their use of violent discipline methods against students. Also, the working conditions often found in schools in sub-Saharan African countries are important sources of teachers’ stress that in turn contributes to their use of violence against students [14, 23]. Work-related stressors including overcrowded classrooms, low wages, insufficient school equipment, work pressure and hierarchical authority structures have been linked to higher levels of perceived stress as well as lower motivation and job satisfaction among teachers in various African countries [24–27]. In addition, students’ emotional and behavioral problems may trigger emotional and physical violence by teachers [10]. Notwithstanding, inadequate training may lead to teachers lacking knowledge about non-violent discipline methods and support strategies for troubled students [28–30], although other evidence suggests that Kenyan teachers were aware of alternative methods, but considered them ineffective [20].

### Effects of violence on child development

Extant research has documented the detrimental short-term and long-term consequences of child maltreatment including physical and emotional violence on children’s development, health and functioning over their life course, including low self-esteem, internalizing problems, e.g. depression and anxiety, externalizing problems, e.g. attention problems and antisocial behavior, substance abuse, suicidality, physical injury and chronic morbidity, impaired cognitive ability, poorer academic performance, lower socioeconomic well-being as well as ongoing victimization and perpetration of violence [31–34]. While most of these studies focused on children and adolescents’ victimization in the family context, recent reviews found comparable negative effects of physical violence by teachers in schools including physical injury and even death, poor academic outcomes, mental health and behavioral problems [5, 10]. The available studies focusing on emotional abuse by teachers have reported similar effects [35–37]. Importantly, the observed associations between exposure to violence by teachers and poorer performance on tests of academic skills, verbal, and educational functioning suggest that violence by teachers may interfere with children’s capacity to learn and thrive at school, thereby contradicting the practice’s intended purpose of improving discipline and school performance [5, 10, 38]. Moreover, the hostile and humiliating environment created by violence in the classroom increases children’s feelings of fear and dislike of school, which may lead them to avoid or even drop-out of school [5, 38]. These detrimental effects translate into enormous costs to societies, e.g., stemming from lower income and productivity and higher expenses for social and health services [5].

### Preventative interventions targeting violence by teachers

The high prevalence of violence by teachers observed across various cultural settings and its detrimental consequences for the individual victims, their families, communities and societies call for joint global and national efforts targeting multiple levels, including legislative reforms prohibiting and sanctioning the use of violence at schools, public education and awareness programs about the negative consequences of violence, the strengthening of structures for reporting the use of violence at schools and the provision of alternative non-violent discipline methods to educators [5, 39]. Global and continental initiatives such as goal 16:2 of the United Nations’ Sustainable Development Goals 2030 [40] and the African Charter on the Rights and Welfare of the Child [41] may pave the way for legal and political changes. However, there is also a high need for preventive interventions at the school-level to reduce the use of violence by teachers and school staff against children and adolescents, particularly in sub-Saharan African countries where violent discipline at schools is both highly prevalent and socially accepted [4, 17, 42]. Notwithstanding, although non-governmental organizations have recently introduced a number of programs in low-and middle-income settings, few of them have been rigorously evaluated in terms of their efficacy to reduce violence by teachers [5, 43].

For instance, in Jamaica, the Irie Classroom Toolbox intervention has been recently evaluated in a cluster-randomized controlled trial in 76 preschools [44]. The intervention does not explicitly target teachers’ attitudes towards violence but aims to reduce violence against children by promoting teachers’ socio-emotional competence and equipping them with positive non-violent discipline techniques. Observations of teachers’ behavior showed that teachers in the intervention group used significantly less physical and emotional violence against children directly after the intervention and at 1-year follow-up compared to teachers in the control group [44]. A small cluster randomized trial of an adapted version of the Irie Classroom Toolbox in 14 Jamaican primary schools further showed that grade 1 primary school teachers in intervention schools used significantly less violence against children than teachers in control schools [45].

In the context of sub-Saharan Africa, the most rigorously evaluated intervention to date is the Good Schools Toolkit, which has been tested in a cluster randomized controlled trial in 42 primary schools in Luwero district in Uganda [2, 46]. The Good Schools Toolkit promotes the use of non-violent discipline techniques through a range of activities implemented at the whole school over an extended period of time. The results of the evaluation trial showed significant reductions in the past-week prevalence of physical violence as reported by students and by school staff in intervention compared to control schools at follow-up [46].

We argue that interventions to reduce violent discipline at schools in sub-Saharan Africa should primarily work with teachers as the ones who actually use violence, focusing on both changing teachers’ attitudes towards violence and providing them with alternative non-violent discipline strategies. Moreover, interventions need to be brief, require relatively few resources and emphasize transfer of intervention content to teachers’ daily work in order to support dissemination in low-income settings. In addition, interventions should be applicable to a wide target group of teachers and students, i.e., different educational stages (primary and secondary education). The intervention *Interaction Competencies with Children – for Teachers (ICC-T)* meets all these criteria. Based on attachment, behavioral and social learning theories, *ICC-T* aims to reduce the use of physical and emotional violence by teachers against students and to improve teacher-student interactions by enabling teachers to learn and practice essential interaction competencies with children [23, 42]. Cluster randomized controlled trials at primary and secondary schools in Tanzania and Uganda have provided initial evidence for the feasibility and effectiveness of *ICC-T* to decrease teachers’ positive attitudes towards violence as well as student- and teacher-reported use of violence against students [3, 4, 42]. In a trial at Tanzanian primary schools, *ICC-T* also led to a reduction in student-reported victimization by peers, suggesting a spill-over effect of the intervention on peer violence [42].

### Aims and objectives

Given the paucity of scientifically evaluated interventions to reduce violence by teachers against children and adolescents in general and in sub-Saharan Africa in particular, we aim to evaluate the effectiveness of the preventive school-based intervention *ICC-T* at primary and secondary/junior high schools in Tanzania, Uganda, and Ghana. In doing so, we aim to consolidate initial evidence on the feasibility and effectiveness of *ICC-T* in primary schools in Tanzania [23, 42] as well as secondary schools in Tanzania [3] and Uganda [4] and to provide a first rigorous evaluation of *ICC-T* at primary school level in Uganda and in different school types in a country outside of East Africa (Ghana). Importantly, the three countries do not only differ in terms of cultural and societal background but also to what extent violence against children is legal. For instance, in Tanzania violence is still legal in all settings, whereas in Uganda and Ghana it is officially not legal at school [47]. However, recent national survey data indicates similarly high prevalence rates of school violence against children in all three countries [48–50]. As the ongoing higher use of violence by teachers irrespective of the legal circumstances can partly be attributed to societal norms and beliefs favouring violent discipline and the lack of knowledge about alternative non-violent discipline methods among teachers, interventions jointly addressing these challenges are likely to be particularly effective in reducing teachers’ use of violence. Therefore, we hypothesize that the implementation of *ICC-T* will reduce the use of physical and emotional violence by teachers across educational settings, societies, and cultures in sub-Saharan Africa. We also expect the *ICC-T* intervention to have a positive impact on children and adolescents’ functioning (i.e., mental health, well-being, academic performance).

## Methods

### Design

**Fig. 1:**
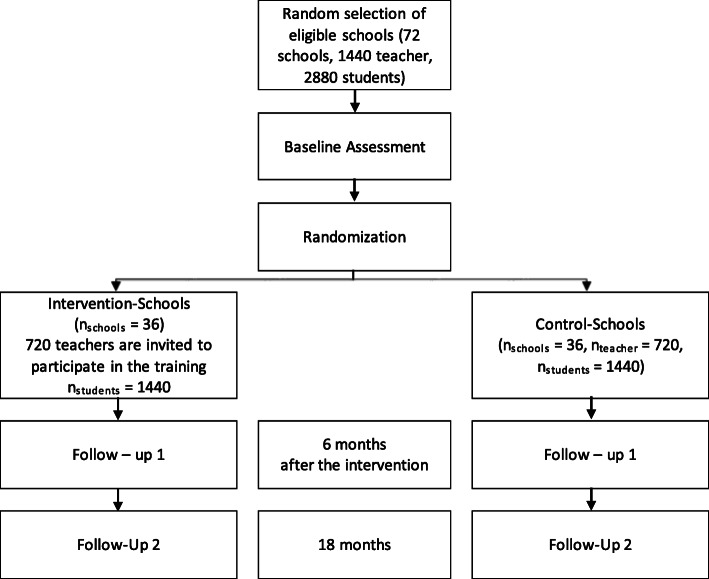
Flow charf of the study design

**Fig. 2:**
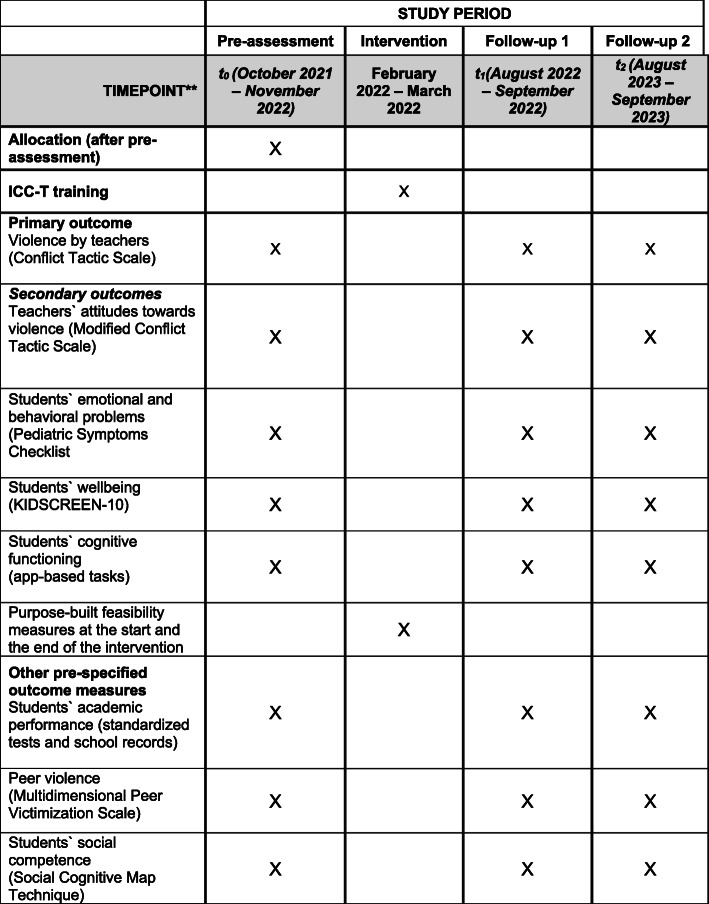
Study time line

Using a two-arm multi-site cluster randomized controlled trial (MSCRCT), this study will include a total of 72 schools (24 schools in each country: Tanzania, Uganda, and Ghana). Half of the schools [36] will be randomly allocated to the intervention group, which will receive the ICC-T intervention, and the other half to the control group, which will receive no intervention. The study will adopt a longitudinal design and involve three data collection phases: baseline assessment directly before the intervention (t0) and two follow-up assessments approximately 6 months (t1) and 18 months (t2) after the intervention (see study flowchart in Fig. 1 and timeline in Fig. 2).

### Study setting and sampling

The study will be carried out in public primary and secondary/junior high schools in Tanzania, Uganda, and Ghana. A multi-stage sampling procedure was applied to ensure a sample of schools that can be considered representative for each country in terms of geographical, socio-economic, and political aspects. At each stage, the respective sampling units (zones, regions, districts) were weighed by their number of schools according to probability-proportional-to-size sampling. First, three administrative zones were randomly selected in each country. Next, one region in each of the selected zones in each country was randomly selected. In the next step, one district in each of the chosen regions in each country was randomly selected. The selected zones, regions and districts per country are displayed in Table 1.

**Table 1:** Selected zones, regions and districts per country

Country	Zone	Region	District
Tanzania	Eastern/Coastal	Lindi	Kilwa District Council
	Lakes	Mwanza	Mwanza City Council
	Southern Highlands	Iringa	Iringa District Council
Uganda	Central	Buganda	Kayunga District
	Western	Toro	Kasese District
	Northern	Acholi	Pader District
Ghana	Southern	Central	Mfantseman Municipality
	Middle	Bono East	Techiman Municipality
	Northern	North East	Bunkpurugu District

*Notes:* Three zones each were randomly selected from the six zones in Tanzania (Northern, Eastern/Coastal, Central, Lakes, Southern Highlands, Zanzibar) and from the four zones in Uganda (Central, Western, Eastern, Northern). As a geographical division into three belts (Southern, Middle, Northern) is common in Ghana, each belt was included at this sampling stage. In Uganda, the region Buganda is equivalent to the Central zone

#### Schools

**Fig. 3:**
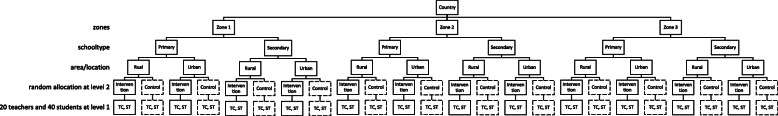
Sampling procedure in each study country. Note. Country*: the flowchart presents the selection procedure for only one country, but it applies to all project countries (Tanzania, Uganda and Ghana). The term “secondary” also includes junior high schools

In each of the selected districts, schools meeting the following criteria will be eligible for inclusion into the study: 1. Public, day-care and mixed-gender primary and secondary/junior high schools. 2. At least 40 students in the selected class/stream (class 3 in primary and form 1 in secondary/junior high school). In case a selected school has less than 40 students in a class or stream, it will be combined with a neighboring public school (within 15 km) to a school cluster and 20 students from each school will be selected. 3. At least 15 and no more than 50 teachers at a school. In case of less than 15 teachers, a school cluster with a neighboring school (within 15 km) will be formed and all teachers officially working at these schools will be included. The upper limit of 50 teachers is due to practical difficulties related to providing the intervention to a higher number of potential participants. Official lists of available schools will be obtained from the relevant authorities and schools will be stratified based on school type (primary vs. secondary/junior high) and whether the school is in an urban or rural setting. The latter will be determined using the database Africapolis (www.africapolis.org), which defines an agglomeration as urban if it constitutes a continuously built-up area with less than 200 m between buildings and its population exceeds 10.000. In the case of entirely urban districts, e.g., Mwanza in Tanzania, existing official classification on the ward level will be used. After listing the stratified schools in each district in alphabetical order, four primary (two urban and two rural) and four secondary/junior high schools (two urban and two rural) will be randomly selected in each of the three districts per country, implying a total of 72 schools (24 per country). The stratified randomization based on school type and urban/rural location results in 36 sites or school pairs, in which one school will be randomly allocated to the intervention group and one school to the control group (see Fig. 3). All random selections of country zones, regions, districts, and schools as well as the allocation of schools to the two study conditions are performed by an independent researcher neither belonging to the core research nor the data collection teams.

#### Participants

In Uganda and Tanzania, primary and secondary school takes 7 and 4 years respectively, while in Ghana, primary school takes 6 years and junior high school takes 3 years. Due to the longitudinal nature of the study with three data collection points spanning across up to 2 years, primary school students in their 3rd year (approximated age: 8–9 years) and secondary/junior high school students in their 1st year (approximated age: 12–14 years) will be included in the study. Students must be below the age of 18 at the start of the study to be eligible for participation. On the primary school level, the inclusion of children in the 3rd year will ensure their sufficient abilities to comprehend interview questions and their availability for follow-up assessments as they will not shift to a consecutive school form.

We conducted an a priori power analysis using the software Optimal Design [51], considering the nested study design. A previous effectiveness trial of *ICC-T* in Tanzanian secondary schools had shown moderate and large effects on student-reported and teacher-reported violence by teachers respectively [3]. In this larger effectiveness trial, we expect small detectable effects of 0.20 standard deviations (SD) for student-reported violence and of 0.25 SD for teacher-reported violence. Based on previous and ongoing trials of *ICC-T* [3, 4, 42] as well as similar school-level interventions [45, 46], we further expected an intra-class correlation coefficient (ICC) of .05 for student-reported and of .10 for teacher-reported violence. In previous trials, the covariance between baseline and follow-up scores of violence at the school level was 0.30, which is also considered in the power analyses. Assuming no dropout at the school-level, a conservative estimate of 40 students per school and an ICC of .05, our study will have 80% power to detect a reduction of 0.20 SD in the primary outcomes of student-reported violence by teachers in intervention schools at a 5% level of significance. The total required sample of students is thus *n* = 2880, considering a potential drop-out rate of 30% through the study course. Depending on the school type, 40 students from the 3rd year of primary school or 40 students from year one of secondary/junior high school will be stratified by gender and randomly selected from class lists provided by the school administration. We will include all teachers officially working in the selected school or school cluster. With no drop-out at the school level, an average number of 20 teachers per school and an ICC of .10, our study will be powered at 80% to detect a reduction of 0.25 *SD* in the primary outcome of teachers’ self-reported use of violence against students in intervention schools at a 5% level of significance. This requires a total sample of teachers of *n* = 1440, allowing for a drop-out rate of about 25% during the study. We assume the power calculation to be conservative since some variation between the schools may be explained through blocking by school type and location.

### Procedures

Before data collection, the research team consisting of psychologists from Bielefeld University and the respective partner universities in Tanzania (Dar es Salaam University College of Education), Uganda (Mbarara University of Science and Technology) and Ghana (Presbyterian University College Ghana) will select fifteen research assistants in each country and train them in data collection in a one-week workshop. The research assistants are required to hold or currently pursue a university bachelor’s degree, to be fluent in English and the local language and to have prior experience in research projects on social/health-related matters. The assessors will be blind to the allocation of the schools to the intervention and control groups. The assessment of students consists of a structured interview, a cognitive testing, and an assessment of academic performance and will take about one and a half hours. The assessment of teachers consists of a structured interview and takes about 1 h. In the interview, assessors will directly enter participants’ responses into Android tablets using the survey software *SurveyToGo* [52]. The cognitive testing will also be administered to the students through the tablets, while the academic performance test will be administered in a paper-pencil format. All measures will be administered with standardized introduction and administration procedures to ensure high objectivity and reliability during data assessment. Following established scientific guidelines [53], all instruments will be translated from English to the respective ethnic local language by independent translators and then back to English by different translators. The back-translated instruments will then be compared with the original instruments to ensure correct translation and equivalence of the content. All interviews will be preferably conducted in the local language to ensure participants’ full understanding, with the option to conduct the interview in English, for example if the local language is not the participant’s mother language.

Prior to data collection, selected students will receive a letter explaining the study aims and procedures together with an informed consent form to their parents to seek parental consent. Students whose parents have signed the informed consent form will be invited to an interview in a quiet and discrete setting in the school premises. Before the interview, each student will be given detailed written and oral information on the study procedure, the confidentiality of their data, and their right to withdraw from the study at any time without any consequences. The interview will only be conducted if primary school students provide their oral assent and secondary/junior high school students provide their written consent. Structured interviews with students will be conducted by assessors who have received specialized training in the assessment of children and adolescents. The assessment procedure will be repeated in the same way at 6-months and 18-months follow-up. Students can be considered masked throughout the study as the intervention only targets teachers.

After being introduced to the study in a formal information session, all teachers at the selected school will be invited to participate in an interview. Teachers willing to participate will receive detailed written and oral information on the study procedure, the confidentiality of their data, and their right to withdraw from the study at any time without any consequences. Upon providing informed consent, the interview will be conducted in a quiet and discrete setting within the school setting. The assessment procedure will be repeated in the same way at 6-months and 18-months follow-up. Given the nature of the intervention, teachers are masked at baseline assessment, but unmasked at the follow-up assessments.

### Intervention description

The ICC-T intervention consists of a 5.5 days (8 h on a full day) training workshop for teachers. The ICC training concept is based on the childcare guidelines of the American Academy of Pediatrics [54] and has so far been adapted and initially evaluated for caregivers working in institutional care settings (*ICC-C* [55];) and teachers working in primary [23] and secondary schools [3]. *ICC-T* aims at preventing harsh and violent discipline in the school setting and improving teacher-student relationship by changing teachers’ attitudes towards the use of violence and enabling them to learn non-violent discipline strategies. The implementation of *ICC-T* is guided by four key principles: First, a participative approach encourages teachers to actively contribute to the training. Second, intensive practice is combined with theoretical input to enable teachers to integrate the acquired skills into their daily work routine at school. Third, a trustful atmosphere during the workshop assures confidentiality and invites participants to share and reflect upon their work-related problems, needs and personal experiences with violent discipline. Fourth, sustainability of the training is ensured through various activities including intensive practice and repetition of the content, self-reflection of personal behaviour, team-building measures, organisation of peer consultation and referral networks as well as ongoing support supervision.

The sessions of the *ICC-T* training workshop focus on five core components that foster positive teacher-student relationship, reduce teachers’ use of violent discipline and ultimately improve children’s wellbeing: 1) Sessions about teacher-student interactions aim to promote teachers’ empathy and understanding of their students’ behaviour and to raise teachers’ awareness of being a role model for students. 2) Sessions on maltreatment prevention aim to raise teachers’ awareness of the negative consequences of violent discipline on children’s well-being by inviting teachers to reflect on their own experiences of violence as a child and connect these experiences and associated feelings to the causes and consequences of their current violent behavior. This component is closely linked to the 3) sessions on effective discipline strategies, which aim to equip teachers with non-violent behavioral skills and tools helping them to maintain and reinforce desired behaviors and to change undesirable behavior by students. 4) Sessions on identifying and supporting burdened students intends to raise teachers’ awareness for common internalizing and externalizing problems among students and to increase their ability to identify and adequately support students with these problems. 5) Sessions on implementation aim at integrating the learned knowledge and skills into everyday school life and at ensuring sustainability by establishing support networks such as peer consultation and collaboration with school counsellors.

### Intervention procedures

The *ICC-T* intervention will be implemented in the selected schools by trained facilitators with a background in psychology and/or teaching. Participation in the training workshop will be free of charge. Participating teachers will be provided food and drinks as well as transport compensation of approximately 4$ per day. All teachers at a selected school will receive detailed written information on the training procedure, the voluntary nature of their participation as well as their right to withdraw from the training at any point. Teachers who agree to participate in the training will be asked to sign an informed consent form. Confidentiality of participants’ personal data and information shared during the training will be ensured at any time.

Treatment fidelity will be monitored in several ways. After each session, both facilitators will fill out a short purpose-built questionnaire including items on the session’s duration, applied methods, and perceived uptake of the session content by participants as well as a checklist on possible deviations from the intervention manual and didactical aspects. Moreover, at the end of each workshop day, four randomly selected participants will be asked to fill out a purpose-built questionnaire on their perceived understanding of that day’s training content and the helpfulness of the applied methods in delivering the content. In addition, all participants will be asked to evaluate the training contents and methods using a purpose-built questionnaire at the end of the workshop. Finally, two independent raters will evaluate video and audio recordings of pre-determined sequences of approximately 10 min to determine whether intervention workshops were implemented in line with the manual.

### Control

No intervention will be implemented in control schools. The research team will be in close contact with the control schools to ensure that no similar intervention will take place at the schools during the study. Apart from the intervention, all data collection procedures at baseline and follow-up assessments will be implemented in control schools in the same way as in intervention schools.

### Outcome measures

Our study aims to test the effects of *ICC-T* on teachers’ use of violence in primary and secondary/junior high schools in Tanzania, Uganda, and Ghana. This primary outcome will be assessed by students’ self-reported experiences of violence by teachers as well as teachers’ self-reported use of violence against students. Secondary outcome measures include children’s self-reported emotional and behavioral problems, quality of life as well as students’ cognitive functioning. Additional outcomes will be students’ experiences of peer violence, social competence and their educational performance assessed through standardized literacy and numeracy tests and grade records provided by the school administration. All outcomes will be assessed using measures that have been used in previous studies in sub-Saharan Africa with acceptable to good psychometric properties. Measures of cognitive and academic outcomes will be adapted to and pilot-tested in the specific study contexts.

#### Children

##### Exposure to violence by teachers

Students’ experiences of physical and emotional violence by teachers will be assessed using the Conflict Tactic Scale (CTS [56];). The original CTS covers various methods adults use to manage conflictual situations with children including physical assault, psychological aggression, non-violent discipline, and neglect with 27 items. In the current trial, an adapted version of the CTS including 16 items on experienced physical violence, 7 items on experienced emotional violence and 3 items on witnessed violence by teachers will be used. The items are answered on a 6-point Likert scale from 0 (this has never happened) to 5 (more than 10 times) and will be asked referring to the past week. Subscale scores are derived by summing up all item scores. The CTS has been implemented in previous studies in East Africa to assess students’ experiences of violence by teachers and has demonstrated acceptable psychometric properties [14, 21, 57].

##### Mental health problems

The Pediatric Symptom Checklist – Youth Report (PSC-Y [58];) will be used to assess children’s emotional and behavioral problems. The PSC-Y consists of 35 items rated on a 3-point Likert scale from 0 (*never*) to 2 (*often*), which can be summed up to a total score of emotional and behavioral problems ranging from 0 to 70. Factor-analyses of the parent- and youth-report version of the PSC revealed a 3-factor structure of internalizing problems, externalizing problems and attention problems [59, 60]. Adapted versions of the PSC haven been used with HIV-infected children in Botswana [61] and school children in Uganda [62] with good psychometric properties, indicating the instrument’s applicability in the sub-Saharan African context.

##### Quality of life

The KIDSCREEN-10 [63] will be used to assess children’s perceived quality of life. The KIDSCREEN-10 conceptualizes quality of life as a multidimensional construct covering physical, emotional, social, and behavioral aspects of well-being and functioning. Children answer the 10 items referring to the past week on a 5-point Likert scale ranging from 0 (*not at all)* to 5 (*extremely*). Having been extensively used in clinical and epidemiological studies in Europe, North and South America, Africa and Asia, the KIDSCREEN-10 has cross-cultural validity to assess children’s and adolescents’ self-reported quality of life [63].

##### Cognitive functioning

We will use four classical tasks implemented in the Android application Psych Lab 101 [64] to assess different aspects of children’s cognitive functioning: A visual search task to assess children’s selective attention, a numerical Stroop task to assess children’s ability to resist interference by distracting information, a delayed match-to-sample task to capture children’s working memory and a continuous performance task to assess children’s impulsivity. These tasks were chosen because they are independent of language and they cover “core” cognitive abilities that have been shown to be affected by exposure to maltreatment [65, 66]. Prior to data collection, we will conduct a pilot-assessment to ensure feasibility of the tablet-based assessment.

##### Social competence

We will assess children’s social competence in two ways. First, we will assess children’s social status in their peer networks using a well-established peer-nomination procedure, the social cognitive map (SCM) technique [67]. This procedure asks children to name a group of children in their class to which they belong as well as other groups of friends in their class. Based on the number of nominations as members of a group, the social centrality status of individual children can be determined [68]. Moreover, children are asked to nominate three classmates they like most and three they like least, which yields an indicator of social preference status for each child. The SCM technique makes it possible to reliably identify social groups with proportions of respondents from a social network as small as 50% [68]. The technique has been successfully implemented in a previous study with primary school children in Tanzania [69]. Second, we will use the 8-item short form of the PROMIS pediatric peer relationship scale [70] to assess the quality of children’s relationships with peers and friends through their self-report. The items are rated on a 5-point Likert scale from 0 (*never)* to 5 (*almost always*) and refer to the past 7 days. The scale comes with good psychometric properties and has been used in various different cultural settings including a sample of child patients in Malawi [70, 71].

##### Educational performance

We will assess children’s educational performance in two ways. First, we will use a standardized test of children’s numeracy and literacy skills. Different test versions will be applied with primary school and secondary/junior high school children respectively to consider differences in levels of acquired skills and comprehension between the two age groups. The test for primary school children is based on standardized tests of numeracy and literacy skills developed by the Uwezo initiative [72]. These tests have been applied in large-scale surveys in Kenya, Uganda, and Tanzania to assess learning outcomes of primary school children. The same test will be used across all sites in Ghana, Uganda, and Tanzania, but some of the literacy tasks will be translated into the respective local language. In the absence of brief and contextually appropriate standardized tests of numeracy and literacy skills of secondary/junior high school children in sub-Saharan Africa, we will use a purpose-built test for this age group in our study. The included tasks will focus on essential numeracy and literacy skills independent of national curricula and will be evaluated by educational experts in the different study countries. The same test will be administered to all students and all literacy tasks will be in English as this is the language of instruction at the secondary/junior high school level in the three countries. Both the primary school and secondary/junior high school tests will be pilot tested in each country prior to data collection. As a second indicator of students’ educational performance, we will record students’ scores in core subjects (e.g. Mathematics, English, Science) in the last term exam from the school administration.

##### Peer violence

We will assess children’s experiences of violence by peers using the 24-item version of the Multidimensional Peer Victimization Scale (MPVS-24; 73), which assesses the six subtypes physical victimization, verbal victimization, social manipulation, attacks on property, electronic victimization and social rebuff with four items each. The original 16-item and the 24-item version of the MPVS have shown good psychometric quality [73]. We will additionally assess sexual victimization by peers using four items from the adolescent version of the Sexual Experiences Survey [74]. Two items each will cover sexual harassment and sexual assault by peers.

#### Teachers

##### Teachers’ use of violence

We will use a modified version of the CTS to assess teachers’ use of physical (16 items) and emotional violence (7 items) against students in the past week. The teacher version uses the same answer scale and scoring as the child version (see above for more details). The CTS has proven its usefulness and feasibility as a measure of teachers’ self-reported use of violence in the classroom in randomized controlled trials [3, 4, 42] and observational studies [14, 21, 22] in Eastern Africa.

##### Teachers’ attitudes towards violent discipline

We will use an adaptation of the CTS to assess teachers’ positive attitudes towards the use of physical and emotional violent discipline. Each item is formulated as a statement beginning with *“When students do something wrong, I think it is OK to …*” and ending with the respective act of physical or emotional violence. The items are answered on a 4-point Likert scale from 0 (never OK) to 3 (always or almost always OK). Subscale items are then summed up to yield scores for attitudes towards physical violence (range 0–48) and towards emotional violence (range 0–21). The modified CTS has been used to assess teachers’ self-reported attitudes towards violent discipline in Tanzania [3, 22] and Uganda [21].

##### Purpose-built measures for ICC-T training evaluation

As this study will be the first implementation of ICC-T on primary school level in Uganda and on any level in Ghana, we will also evaluate the feasibility of ICC-T in these contexts adopting the purpose-built measures as used in previous studies [3, 23] and following the guidelines for feasibility studies by Bowen et al. [75]. In particular, the applicability of the training, i.e., participants’ expectations about the workshop and its relevance in their daily work will be assessed before and directly after the intervention as well as at each follow-up. In addition, the acceptability of the training, i.e., satisfaction with the training and evaluation of new knowledge, and the integration of ICC-T core elements in their daily work will be assessed after the intervention and at each follow-up.

### Measures against bias

Several measures will be taken to minimize the risk of bias and to increase the validity of the findings. First, the stratified random sampling procedure will counteract selection bias. Second, the thorough training of data collectors and the structured interview assessment using carefully selected and contextually appropriate instruments will reduce participants’ reporting biases and increase the validity of responses. Third, as the allocation to intervention and control group will be executed at the cluster level and by the core research team following baseline assessment, those collecting data will be blind to the treatment conditions of the schools. Fourth, while teachers’ reports of violence against students are likely to be biased in the same direction as the intervention effect, the use of students’ reports of violence will provide a conservative test of the intervention effect [46]. Fifth, analyses will be carried out based on the groups as randomized (“intention to treat”) to avoid incomplete accounting of participants and outcome events.

### Ethical considerations

As this research focuses on violence against children, ethical considerations and child protection are essential. The study has obtained ethics clearance from the Ethics Review Boards of Bielefeld University (No. EUB 2019–165) in Germany; the National Institute for Medical Research (No. NIMR/HQ/R.8a/Vol. IX/3381)) and Commission for Science and Technology (No. 2020–204-NA-2020-96) in Tanzania; Ethical Review Board of Mbarara University of Science and Technology (No. 17/03–20) and the Ugandan National Council for Science and Technology (No. SS444ES) in Uganda; and the Ethics Review Board of the Presbyterian University College Ghana in Ghana. To protect participants’ identity, a pseudonymization procedure will be applied by assigning a numeric code to each participant a priori. Participants’ data will be stored only together with their respective code in a password-protected folder on a secure server accessible only to the study investigators. The document linking the numeric codes to individual participants will be kept strictly confidential and separate from other data in a specific encrypted and password-protected file that will only be accessible to one pre-assigned research team member in each country who does not have access to pseudonymized data. This also refers to video and audio recordings of teachers participating in the intervention. Personal data will not be disclosed to any other person without the participant’s permission or as required by the law.

Behavioral intervention studies are minimum risk studies and we do not expect any adverse events as a consequence of the intervention itself. However, in case of any unexpected adverse effect, the researchers will document and report such occurrences to a trained psychologist on the research team. In case the problem is severe, the psychologist will report the problem to an independent monitoring and advisory board consisting of four experienced researchers within 1 week. Questions about experiences may evoke upsetting memories if the participant experienced similar events in his or her life. Participants who will experience any psychological distress during the data collection will be provided with psychological support by the trained psychologist on site. For participants who experience adverse or unexpected events, appropriate referrals and follow-up for specialized services and further management will be made on a case-by-case basis. The trial is overseen by a monitoring board, which ensures that the collection and management of data comply with ethical standards at any time.

### Data analyses

Baseline assessment data will be used to provide information about the prevalence of maltreatment and violence in different settings as well as children’s mental health and well-being. Longitudinal analysis will be carried out based on the groups as randomized (intention to treat). As drop-outs and missing data at follow-up assessment are likely given the longitudinal study design, we aim to apply full information maximum likelihood estimation to obtain unbiased parameter estimates. In our main analyses, we will investigate the effect of the intervention on the primary and secondary outcome measures in comparison to the control group. Due to the naturally nested data structure, we will apply multilevel analyses. Latent growth modeling or cross-lagged path models will be used to estimate the directional influence of violence by teachers on primary and secondary outcome variables over time. Results will be presented including appropriate effects sizes and with a measure of precision (95% confidence intervals). Effect size η^2^ ≥ 0.01, η^2^ ≥ 0.06 and η^2^ ≥ 0.14 will be considered to represent a small, moderate, and large effect, respectively [76].

## Discussion

The exposure to violence by teachers places children at risk of developing mental health problems, psychosocial and academic difficulties and thus contributes to a loss of social and human capital on a community and society level [5]. Considering that the prevalence of violence against children at school is particularly high in low- and middle-income countries, the prevention of violence by teachers may be an important element in efforts to foster socio-economic development in these countries. Studies conducted in sub-Saharan African countries indicate that physical and emotional violence by teachers and school staff characterize students’ school life, including those where violence has officially been banned from schools [10]. This suggests that legal measures may be necessary, but not sufficient, to end children’s victimization by teachers. Cultural norms, beliefs, and attitudes endorsing violence as an effective means of managing students’ behavior as well as a lack of non-violent discipline strategies are likely to contribute to the widespread ongoing use of violence by teachers in sub-Saharan Africa.

Compared to legal and structural factors including poor working conditions, teachers’ attitudes and specific behaviors may be more readily modified by prevention programs. This is also in line with the idea of a “bottom-up” approach towards the prevention of violence, which considers schools as engines for societal change [77]. Notwithstanding, there is currently a dearth of scientifically evaluated school-based interventions that address these factors to reduce violence by teachers against students. The current study therefore aims to evaluate the effectiveness of *Interaction Competencies with Children – for Teachers (ICC-T)* at primary and secondary/junior high schools in Tanzania, Uganda, and Ghana using a MSCRCT design.

Drawing on attachment and social learning theories and combining intensive practice with discussions in trustful and confidential settings, *ICC-T* aims to achieve change through two key mechanisms that complement each other. On the one hand, self-reflections about teachers own experiences of violence, discussions, role plays and theoretical input aim at increasing teachers’ empathy with students, thereby enabling them to visualize the connection between violence and its negative consequences and facilitating a change of attitudes towards violent discipline. On the other hand, teachers are equipped with a repertoire of non-violent action skills and strategies to handle everyday situations in their classroom. Teachers intensively practice these strategies in role plays and actively elaborate ways how to integrate them into their daily work. By targeting *both* attitudes towards the use of violence *and* alternative non-violent strategies, we expect ICC-T to achieve a sustainable reduction of teachers’ use of physical and emotional violence against students.

Previous trials of *ICC-T* have provided initial evidence for its feasibility and effectiveness at primary and secondary schools in Tanzania [3, 42] and at secondary schools in Uganda [4]. Like those studies, the current study will adopt a two-arm cluster randomized controlled trial design. However, it will extend the previous trials by including a larger number of clusters and a longer follow-up period of 18 months and by considering an additional educational setting (primary schools in Uganda) and cultural context (Ghana). In so doing, the study will provide a particularly strong test of the effectiveness of ICC-T and its generalizability across educational systems, countries, and cultures. Moreover, the longitudinal and experimental design including the controlled manipulation of violence by teachers through ICC-T will yield insights into temporal and causal associations between children’s exposure to violence and their mental health, psychosocial, cognitive, and academic functioning. The use of nationally representative samples of students and teachers in each country will inform about prevalence rates of violence in the school setting. To quantify students’ exposure to violence, we will not only rely on teachers’ reports, which may be biased in the direction of the intervention effect, but also on students’ self-reported exposure to violence, which can be considered a more conservative test of the intervention effect. The use of structured interview assessment and standardized cognitive and academic performance tests are likely to strengthen the validity of findings by reducing reporting and common-method bias.

Notwithstanding, the study has some limitations. Due to the longitudinal and experimental nature of the study, attrition among participating students and teachers may occur. Reasons for attrition include possible transfer from one school to another, absenteeism, or a wilful decision to drop out. Although our power analysis considers individual attrition to a certain extent, we aim to keep it at a minimum. In a similar vein, we do not expect attrition on the school-level, which may nonetheless occur. We aim to minimize variation between study sites through standardized assessment and intervention procedures and through stratification based on school type and location. However, between-country differences may still account for considerable variation between schools. Furthermore, there are strong socio-cultural factors, attitudes, and beliefs that support the use of violence against children. The expected changes in attitudes and behavior can thus be considered only preliminary.

Despite these challenges and limitations, we believe that this study will significantly contribute to the emerging evidence base on the feasibility and effectiveness of school-level interventions to reduce teacher violence in low and middle-income settings in general and of *ICC-T* in particular. Furthermore, the study will contribute to Pan-African [41] and global campaigns [9, 78] to end all violence against children. Being a low-cost and easily applicable intervention, we believe that *ICC-T* will be of great interest to governments, non-governmental organisations, donors, and policy makers in sub-Saharan African countries and beyond. We hope that a successful evaluation of *ICC-T* across educational systems, countries and cultures will convince relevant stakeholders to scale up the intervention on a regional and national level and to integrate it in regular teacher training programs.

## Data Availability

All study materials and the datasets generated and/or analyzed during the current study will be available from the corresponding author on request.
